# Structural and electronic properties of CdTe_1-x_Se_x_ films and their application in solar cells

**DOI:** 10.1080/14686996.2018.1497403

**Published:** 2018-10-01

**Authors:** Martina Lingg, Annina Spescha, Stefan G. Haass, Romain Carron, Stephan Buecheler, Ayodhya N. Tiwari

**Affiliations:** Laboratory for Thin Films and Photovoltaics, Empa - Swiss Federal Laboratories for Materials Science and Technology, Duebendorf, Switzerland

**Keywords:** CdTe_1-x_Se_x_, thin-film solar cells, doping, photovoltaics, 50 Energy Materials, 209 Solar cell / Photovoltaics, 306 Thin film / Coatings

## Abstract

The performance improvement of conventional CdTe solar cells is mainly limited by doping concentration and minority carrier life time. Alloying CdTe with an isovalent element changes its properties, for example its band gap and behaviour of dopants, which has a significant impact on its performance as a solar cell absorber. In this work, the structural, optical, and electronic properties of CdTe_1-x_Se_x_ films are examined for different Se concentrations. The band gap of this compound changes with composition with a minimum of 1.40 eV for x = 0.3. We show that with increasing x, the lattice constant of CdTe_1-x_Se_x_ decreases, which can influence the solubility of dopants. We find that alloying CdTe with Se changes the effect of Cu doping on the p-type conductivity in CdTe_1-x_Se_x_, reducing the achievable charge carrier concentration with increasing x. Using a front surface CdTe_1-x_Se_x_ layer, compositional, structural and electronic grading is introduced to solar cells. The efficiency is increased, mostly due to an increase in the short-circuit current density caused by a combination of lower band gap and a better interface between the absorber and window layer, despite a loss in the open-circuit voltage caused by the lower band gap and reduced charge carrier concentration.

## GRAPHICAL ABSTRACT

**Figure:**
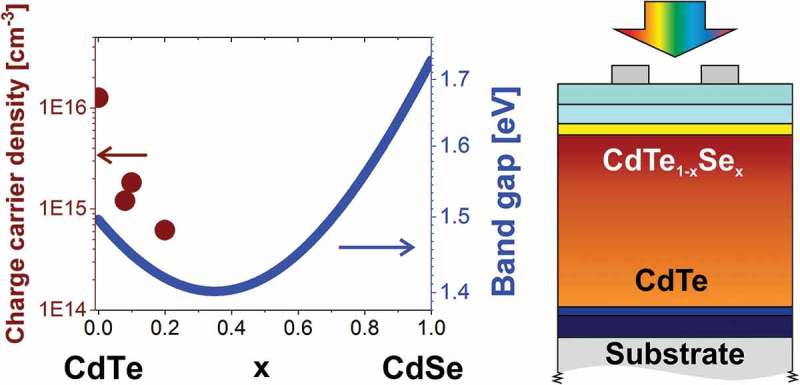

## 1. Introduction

Recent improvements in CdTe solar cell efficiency were mainly reached by increasing the short-circuit current density (*J*_SC_) in state-of-the-art superstrate solar cells. The *J*_SC_ is already close to its theoretical limit [1] and the record cells by First Solar show a potential to go beyond 22% efficiency by modifying the band gap [2]. Further improvements are mainly expected from a higher doping concentration and a longer minority carrier life time. Improvement of both these factors should increase the open-circuit voltage (*V*_OC_). The CdTe_1-x_Se_x_ alloy has been shown to have higher carrier life times than CdTe [3]. In this work, we focus on the other contribution to improving *V*_OC_ by investigating the doping concentrations achievable in this alloy. If the p-type doping can be increased, the quasi-Fermi level for holes is shifted further towards the valence band, thereby improving the *V*_OC_ [4].

The achievable doping concentration in II–VI compound semiconductors is believed to be limited by dopant solubility and formation of compensating defects [5]. Copper is a common dopant used in CdTe solar cells to improve p-type conductivity. First-principle calculations indicate that Cu on a Cd site (Cu’_Cd_) forms deep acceptors [6,7]. However, copper also forms compensating donors on interstitial sites (Cu^•^_i_), and as a result the achievable hole density is limited [6,8]. At low amounts of added copper, the formation of acceptors (Cu’_Cd_) increases the p-type conductivity. At higher amounts of added copper, donor defects (Cu^•^_i_) are formed in addition to the acceptors, reducing the net p-type conductivity. As a consequence, excessive copper amounts are detrimental to the resistivity and the hole density [8]. Additionally, the majority of Cu in solar cells is segregated at grain boundaries [5]. This can influence the minority carrier life time which is limited by recombination at bulk defects and at grain boundaries in the CdTe layer [9].

We investigate here the strategy of alloying CdTe with an isovalent element to change its structural and electronic properties. Alloying CdTe with Se is expected to change its performance as a solar cell absorber material in several aspects [10]. With Se having the same number of valence electrons as Te, substitution of the two does not introduce dopant-like acceptor or donor defects, but it can influence the behaviour of dopants such as Cu. The formation energy of dopants can be changed in the ternary or quaternary alloy compared to the pure binary compound due to electronic and strain effects: For the CdTe_1-x_S_x_ system Ma and Wei predicted a bowing in the formation energy of the Cu’_Cd_ defect, which causes an increased solubility of the defect in the alloy compared to the pure binary constituents [11]. Other effects of alloying are changes in the lattice constant, in the band gap, in the band alignment and in the solubility of dopants. The band gap especially was shown to decrease from 1.50 eV for CdTe to as low as 1.40 eV, potentially increasing the current of a solar cell. Thin film CdTe_1-x_Se_x_ undergoes a phase transition at around *x* = 0.6, from the cubic zinc blende structure of CdTe to the hexagonal wurtzite structure of CdSe. The transition composition can depend on deposition method and temperature [12–14]. We present samples with the zinc blende structure up to *x* = 0.5, with a decreasing lattice constant with increasing *x* that follows Vegard’s law.

CdTe devices in superstrate configuration containing CdTe_1-x_Se_x_ have been investigated before. Poplawsky et al. [12] and Paudel and Yan [15] fabricated CdTe solar cells with CdSe as a window layer, which transformed into a CdTe_1-x_Se_x_ alloy layer due to interdiffusion. This window layer partially or completely replaced the traditional CdS window layer [15]. They showed that the reduced band gap of a CdTe_1-x_Se_x_ window layer translates to a gain in *J*_SC_, but completely replacing the CdS window layer results in a *V*_OC_ loss. Swanson et al. [10] studied solar cells with a CdTe_1-x_Se_x_ layer between a Mg_0.25_Zn_0.75_O window layer and the CdTe absorber layer. They also reported an increase in *J*_SC_ with a reduction in band gap.

In this paper, we investigate the properties of the CdTe_1-x_Se_x_ alloy with a focus on its influence on Cu doping. We measure the achievable charge carrier concentration and its dependence both on the alloy composition and on the amount of added copper.

Based on the results from optical and electrical measurements presented in this paper, we introduce the concept of a substrate configuration CdTe solar cell containing an absorber with compositional grading, which corresponds to grading of structural and electronic properties. Compositional gradings have been used in Cu(In,Ga)Se_2_ solar cells for several years to achieve efficient carrier collection and reduced recombination [16]. More recently, this strategy has also been applied in perovskite solar cells [17].

## 2. Experimental details

To investigate the properties of the CdTe_1-x_Se_x_ alloy, 5–10-µm-thick layers with *x* = 0–0.5 were prepared by co-evaporation in high vacuum of CdTe and CdSe on Corning 7059 borosilicate glass (Corning Incorporated, Corning, NY, USA). The substrate temperature was 350 °C and the CdTe (99.9999%, 5N Plus, Lübeck, Germany) and CdSe (99.999%, Alfa Aesar, Karlsruhe, Germany) sources were independently heated. The growth rates were monitored with two Inficon XTM/2 quartz crystal microbalances. The CdTe_1-x_Se_x_ layers were treated by evaporating 400 nm CdCl_2_ (99.995%, Sigma-Aldrich, Buchs, Switzerland, monitored with a Inficon XTM/2 quartz crystal microbalance [INFICON AG, Balzers, Liechtenstein]) on the layer and subsequently annealing in a tube furnace at 435 °C (25 min) in an oxygen-containing ambient (200 mbar O_2_ + 300 mbar Ar). The layers were doped by evaporation of Cu (Puratronic 99.999%, Alfa Aesar, monitored with an Inficon XTM/2 quartz crystal microbalance deposition monitor) and subsequent annealing in a tube furnace at 400 °C (20 min) in an oxygen-containing ambient (200 mbar O_2_ + 300 mbar Ar). A mask was used for the evaporation of gold contacts for Hall (point contacts in a square geometry with 8 mm side length) and transmission line method (TLM) resistivity measurements (line contacts with 0.2–2 mm distances). The finished layers were annealed at 210 °C for 10 min and then cooled to room temperature within 5 min on a copper plate. The composition of the CdTe_1-x_Se_x_ layers was measured with inductively coupled plasma optical emission spectrometry (ICP-OES, Varian Vista Radial Pro, Agilent Technologies AG, Basel, Switzerland, extraction with HNO_3_ 67 wt% and H_2_O_2_ 30 wt% in water) and energy-dispersive X-ray spectroscopy (EDX, Nova NanoSEM 230 FEI, FEI Europe B.V., Zürich, Switzerland, with Oxford X-Max SDD EDX system, Abingdon, UK) of unpolished and uncoated samples.

Solar cells in substrate configuration were grown on Corning 7059 borosilicate glass. A Mo back contact was deposited by DC magnetron sputtering, followed by 100 nm MoO_3_ (Puratronic 99.9995%, Alfa Aesar) deposited by high-vacuum evaporation monitored with an Inficon XTM/2 quartz crystal microbalance deposition monitor. 5 µm CdTe was evaporated at a substrate temperature of 350 °C. For samples with compositional grading, a CdSe layer was evaporated on top of CdTe by high-vacuum evaporation. In all cases the stack was treated with CdCl_2_ and doped with Cu in the same procedure as described above for the CdTe_1-x_Se_x_ layers on glass. CdS window layers with varying thicknesses were deposited by chemical bath deposition (CBD) at 70 °C, followed by evaporation of 100 nm CdCl_2_ and annealing at 360 °C in an oxygen-containing ambient (240 mbar O_2_ + 260 mbar Ar). On the standard sample without CdSe a second layer of CdS was subsequently deposited by CBD. The front contact ZnO/ZnO:Al was deposited by RF magnetron sputtering, followed by a fork-shaped Ni/Al grid (50 nm Ni/4 µm Al) deposited by electron beam evaporation. The cells were separated by mechanical scribing. The finished cells were annealed at 210 °C for 10 min in air and then cooled to room temperature within 5 min on a copper plate.

The thickness of the CdTe_1-x_Se_x_ layers was measured with a KLA Tencor D-120 Stylus Profilometer (KLA-Tencor, Milpitas, CA, USA). X-ray diffraction in the range 2θ = 10°/20° – 80° using CuK_α_ radiation was performed with a Siemens D5000 X-Ray Diffractometer (Bruker AXS GmbH, Karlsruhe, Germany) with an acceleration voltage of 40 kV to determine the crystal structure and lattice constant. The optical band gap of the CdTe_1-x_Se_x_ layers was analysed by measuring the total optical transmittance and reflectance in the range of 300–1100 nm using a UV-VIS-NIR Spectrophotometer Shimadzu UV 3600 (Shimadzu Schweiz GmbH, Reinach BL, Switzerland) equipped with an integrating sphere. Bulk resistivity measurements were conducted at 25 °C with the transmission line method in 4 terminal sensing. Hall measurements were conducted with an HMS 3000 Hall effect measurement system with the sample temperature controlled between 25–80 °C.

Current density-voltage (J-V) measurements were conducted under standard testing conditions (AM1.5G, 1000 W/m^2^, 25 °C, 4-terminal measurement). The external quantum efficiency (EQE) was measured using a monochromatic light beam chopped at 270 Hz produced with a halogen lamp and a monochromator, under white light bias illumination. The light intensity was calibrated using a calibrated c-Si reference solar cell.

## 3. Results and discussion

### 3.1. Structural and optical properties of CdTe_1-x_Se_x_

**Figure 1.**
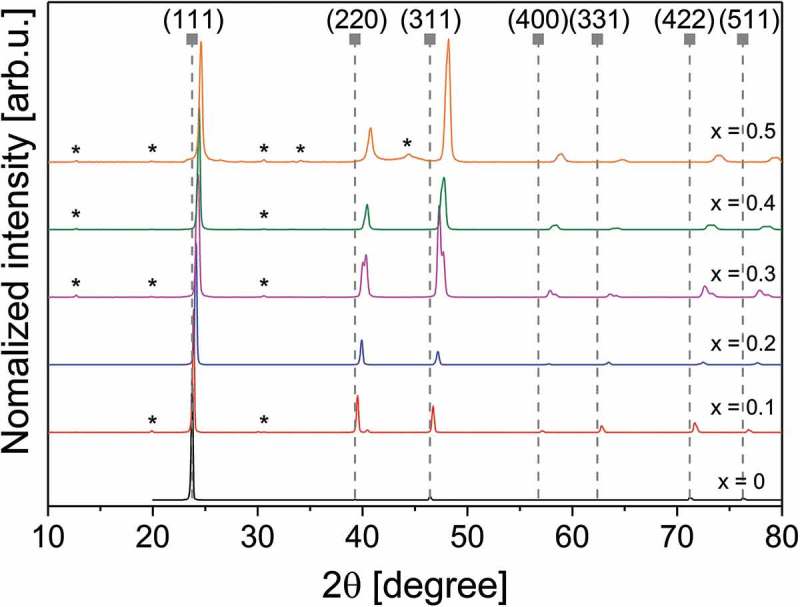
X-ray diffractograms of CdTe_1-x_Se_x_ thin films on glass substrate. The symbols indicate the CdTe reference from [18] (PDF number 01–070–8041).

X-ray diffraction measurements were performed on 5-µm-thick co-evaporated CdTe_1-x_Se_x_ layers (see Figure 1). The structure was identified to be the cubic zinc blende structure of CdTe, which agrees with previous experimental studies [10,18,19]. Secondary phases originating from Te_2_Se_2_O_8_, SeO_2_, and Se_2_O_5_ based phases [20–22] cause Bragg reflections at 2θ angles of 12.6°, 19.9°, 30.6°, and 34.2° in the samples with composition *x* = 0.1, 0.3, 0.4, and 0.5. The sample with *x* = 0.5 exhibits additional Bragg reflexes that could not be assigned yet.

**Figure 2.**
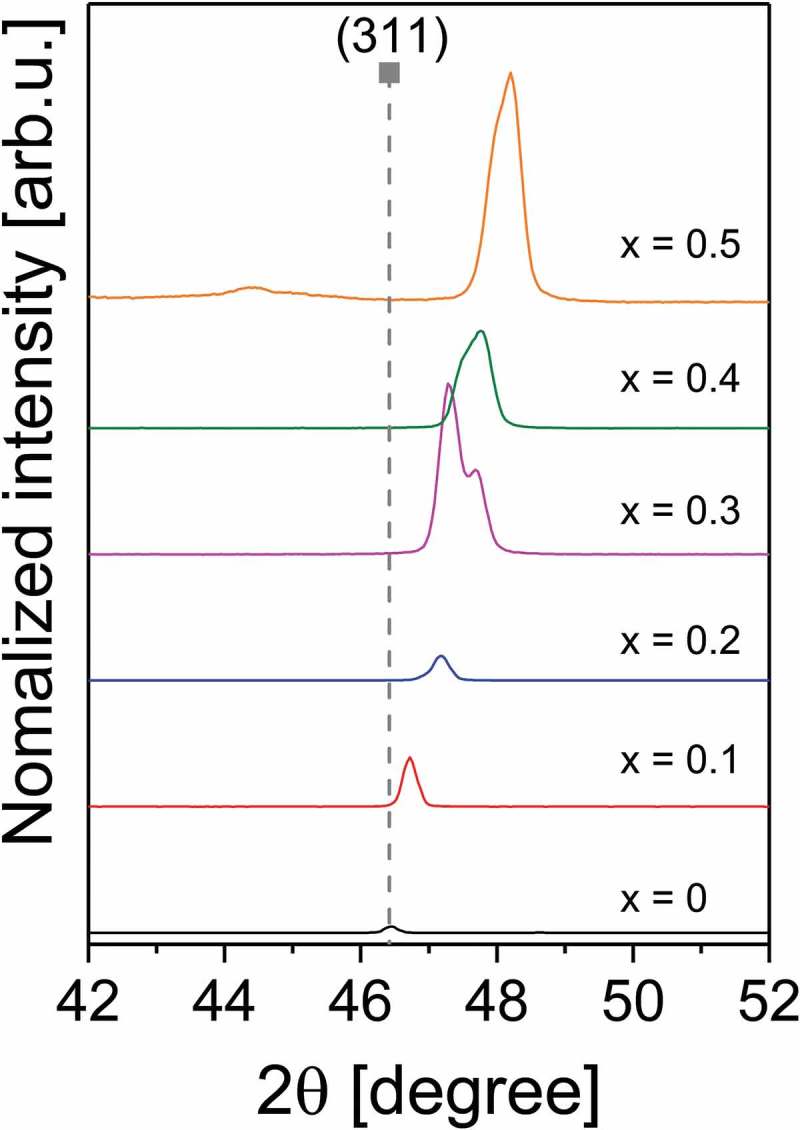
Splitting of the 311 Bragg reflex of CdTe_1-x_Se_x_ thin films on glass substrate. The reference value for the CdTe peak is from [18].

At compositions of *x* = 0.3 and higher, peak splitting occurs in the Bragg reflections at 2θ = 40° and higher (see Figure 2). In literature XRD patterns of CdTe_1-x_Se_x_ no peak splitting was observed [10]. Peak splitting can be an indicator of reduced symmetry, for example a distortion of the cubic lattice in one direction, resulting in a tetragonal crystal structure. This is supported by the fact that the (111) peak is not split. A tetragonal distortion has not been reported for CdTe_1-x_Se_x_. Another possible explanation for the peak splitting is the formation of multiple CdTe_1-x_Se_x_ phases with different chalcogen compositions.

The lattice constants of the samples with *x* = 0–0.2 were calculated from the positions of the seven Bragg reflexes indicated by the reference pattern and by using hkl indices assigned according to crystallographic data from Rabadanov et al [18]. For the samples *x* = 0.3 and 0.4, we assumed a small tetragonal distortion of the lattice. This transforms the (111) Bragg reflex of the zinc blende structure to a (011) Bragg reflex of a body-centred tetragonal structure, and by assuming only a small distortion the lattice constant *c* can be calculated, which is the equivalent to the lattice constant *a* in the cubic structure.

**Figure 3.**
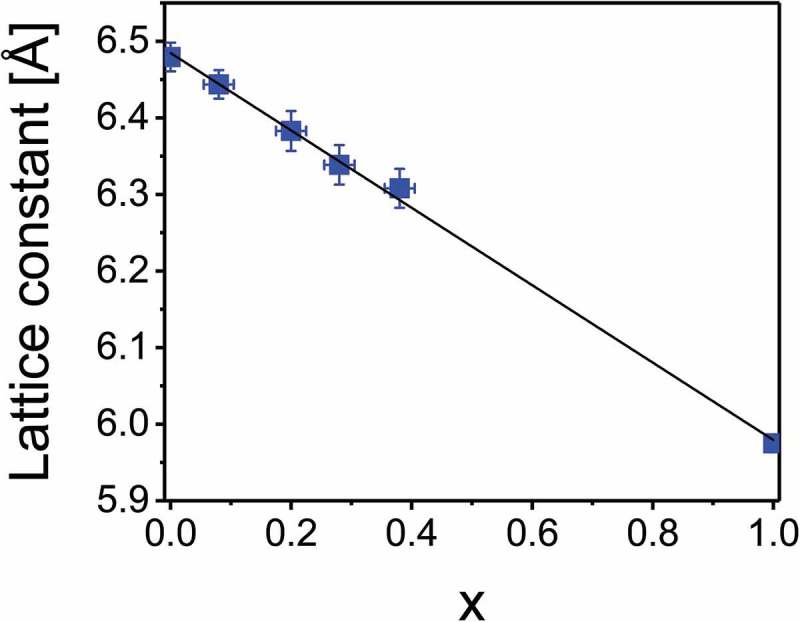
Lattice constant of CdTe_1-x_Se_x_ thin films on glass substrate. The value for zinc blende CdSe (*x* = 1) was taken from literature [23].

The lattice constants calculated from the XRD patterns of CdTe_1-x_Se_x_ with *x* = 0–0.4 are shown in Figure 3, with the value for zinc blende CdSe taken from literature [23]. They follow Vegard’s law with a linear dependence on *x*, with the smaller size of Se compared to Te causing a decrease in lattice constant. The values are in agreement with literature [10,24], reinforcing the hypothesis that the peak splitting is caused by a small lattice distortion and thereby reduced symmetry.

**Figure 4.**
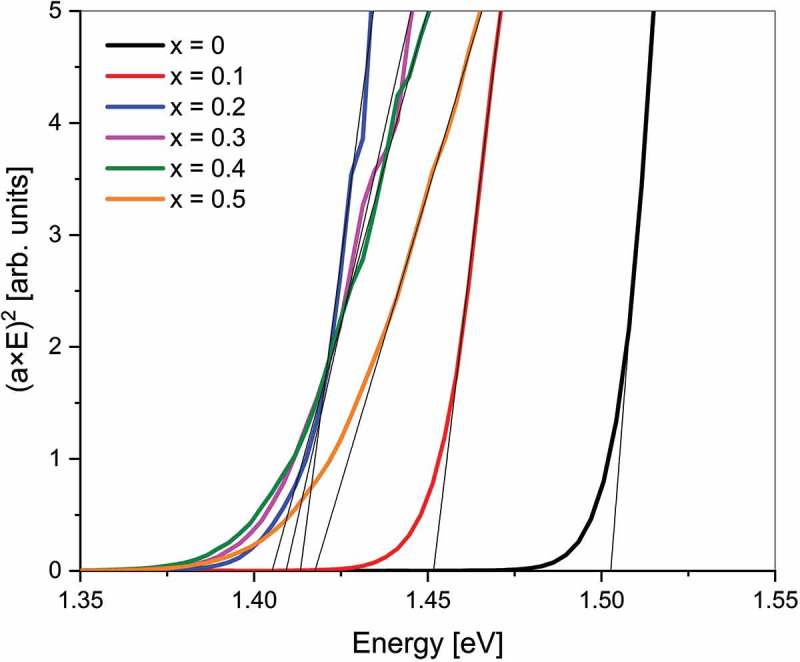
Tauc plots of CdTe_1-x_Se_x_ thin films on glass substrate with linear fitting to extract the band gap.

**Figure 5.**
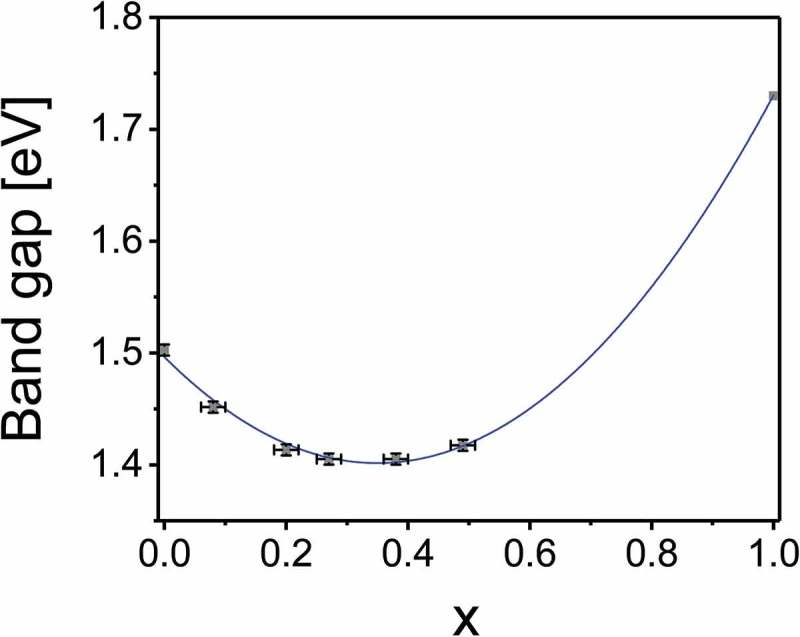
Band gap of CdTe_1-x_Se_x_ thin films on glass substrate determined from the Tauc plot. The band gap value for the pure CdSe (*x* = 1) was taken from literature [25].

The optical band gaps of CdTe_1-x_Se_x_ layers with *x* = 0–0.5 were determined from integrated transmittance and reflectance measurements. The absorption coefficient was calculated with the Beer-Lambert law as $\alpha \left(\lambda \right)=-\frac{1}{d}\cdot \ln\left(\frac{T}{1-R}\right)$, with layer thickness *d*, transmittance *T*, and reflectance *R*. From the Tauc plot ((α*hν)^2^ versus (hν), see Figure 4) the band gaps were extracted as the intercept of the linear fit with the *x*-axis. The values are presented in Figure 5, with the value for *x* = 1 taken from literature [25]. A bowing of the band gap is observed, with a minimum of 1.40 eV at *x* = 0.3 where the band gap value is lower than for either parent compound. The band gap of pure CdSe is known to be 1.74 eV and only marginally depends on the phase [26–28]. A second-degree polynomial fit of the measured values describes the composition dependence of the band gap. The polynomial fit function can be written as

(1) $$E_{G}\left(x\right)=x\cdot E_{G}^{CdSe}+\left(1-x\right)\cdot E_{G}^{CdTe}-0.78\;eV\cdot x\cdot \left(1-x\right)$$

with *E*_G_^CdSe^ and *E*_G_^CdTe^ as the band gaps of CdSe and CdTe, respectively, and the bowing parameter *b* = 0.78 eV. This agrees with the value of *b* = 0.75 calculated from first principles [27] and with experimental bowing parameters from literature that range from 0.59 to 0.92 [24,29]. The band gap of an arbitrary composition x can be calculated using equation (1).

Partial densities of state calculated with a tight-binding method by Tit et al. [29] reveal that the valence band of the CdTe_1-x_Se_x_ alloy consists predominantly of anion states, while the conduction band consists of cation states. They found that the valence band states have lower energies for Se than Te, because Se is more electronegative than Te. This indicates that with decreasing Te content, the local density of states at the valence band edge decreases. The Tauc plots in Figure 4 show a change in slope between *x* = 0.2 and *x* = 0.3, which could be a result of this change in density of state of the valence band edge.

### 3.2. Electronic properties of CdTe_1-x_Se_x_

Dopants with a different size than the host lattice can induce a strain on the structure. We investigate the case of copper, whose size is small compared to Cd. Consequently, a Cu’_Cd_ defect in CdTe puts a strain on the lattice. A change in the lattice constant of the host lattice, as is shown here for CdTe_1-x_Se_x_, can therefore reduce the strain that is induced by such a dopant, and can have an impact on the formation energy of the dopant defect, which in turn influences the solubility of the dopant [11].

The electronic properties of CdTe_1-x_Se_x_ were analysed with resistivity measurements using the transmission line method (TLM) and with Hall effect measurements. CdTe_1-x_Se_x_ layers with *x* = 0–0.2 and varying copper content were investigated. To reduce the influence of grain boundaries on the electrical measurements they were performed on 10-µm-thick layers. The grain sizes scale with layer thickness, therefore the surface area of the grains relative to the surface area of the sample stays constant. As the majority of copper is segregated at the grain boundaries, the amount of copper per grain boundary is independent of layer thickness and we can give the added amount of copper per area instead of per volume of CdTe_1-x_Se_x_.  Before the resistivity and Hall effect measurements, the layers were annealed at 210 °C for 10 min with subsequent rapid cooling on a copper plate (quenching). Previous measurements [5] have shown that quenching decreases the resistivity in CdTe by an order of magnitude probably due to a supersaturation of Cu’_Cd_ in the CdTe bulk. This supersaturation decays exponentially within hours at room temperature [5]. Each sample was quenched separately, the TLM measurements were performed within 2 h after quenching, and the Hall effect measurements were performed within 3 h after quenching.

**Figure 6.**
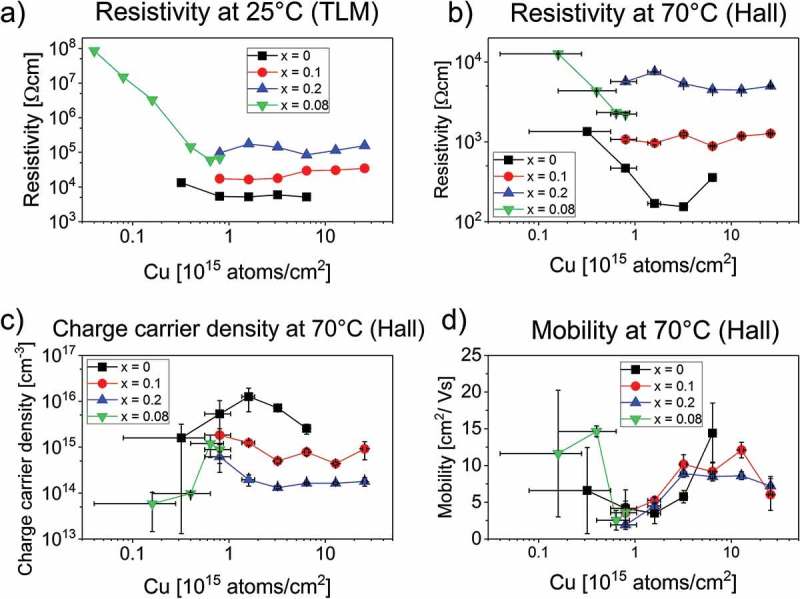
Results from resistivity measurements with the transmission line method at 25 °C (a) and from Hall effect measurements at 70 °C (b–d) of CdTe_1-x_Se_x_ thin films on glass substrate with Cu doping variations for *x* = 0, 0.1, 0.2 and with an additional *x* = 0.08 sample with lower Cu doping.

Figure 6(a) shows the resistivity measured with the transmission line method at 25 °C in darkness. The resistivity of the pure CdTe with different amounts of Cu added is comparable with previously published results [30]. A substantial increase in resistivity is observed if less than approximately 1 × 10^15^ copper atoms/cm^2^ are added. The samples containing Se show a peculiar behaviour. The resistivity of the sample with *x* = 0.1 decreases if less than 5 × 10^15^ Cu atoms/cm^2^ are added. The *x* = 0.2 sample has a maximum in resistivity in the same range of added Cu. In the sample with *x* = 0.08, the resistivity increases by several orders of magnitude if less than 0.5×10^15^ Cu atoms/cm^2^ are added.

Overall, we observe a resistivity increase with increasing *x* in the TLM measurements, with an order of magnitude difference between pure CdTe and CdTe_0.8_Se_0.2_.

Figure 6(b-d) show the resistivity, charge carrier concentration, and mobility determined by Hall effect measurements for CdTe_1-x_Se_x_ layers with *x* = 0–0.2 with a varying amount of copper doping. The measurements were performed at 70 °C in darkness. Due to the high resistivity caused by reduced charge carrier concentration, reliable measurements at lower temperatures were not possible for CdTe samples containing Se.

As a function of copper concentration, a clear minimum in resistivity is observed for pure CdTe in the range of (2–4)×10^15^ copper atoms/cm^2^ added to the surface during the doping step. The charge carrier concentration of this sample is highest with the addition of around 1.6×10^15^ copper atoms/cm^2^.

This is comparable to previous results, where the formation of acceptor defects (Cu’_Cd_) was assumed to be responsible for increasing carrier concentration at low Cu content, and the additional formation of compensating donors (Cu^•^_i_) at higher Cu content reduced the net p-type conductivity [8]. The mobility has a minimum value in the range of (0.5–2)×10^15^ copper atoms/cm^2^, with an increase for higher copper values.

In the Se-containing CdTe samples, little dependence on the copper concentration is observed in the investigated range for the *x* = 0.1 and *x* = 0.2 samples. In the *x* = 0.2 sample, a maximum in resistivity can be seen if around 2×10^15^ copper atoms/cm^2^ is added to the surface during the doping step, comparable to the TLM measurements. With less than 0.8×10^15^ copper atoms/cm^2^ added, the resistivity of the *x* = 0.08 sample increases, in agreement with the TLM measurements (Figure 6(a)).

An increase in charge carrier concentration for Se-containing CdTe samples is observed with decreasing copper concentrations with less than 3×10^15^ copper atoms/cm^2^ added. With addition of less copper than 0.6×10^15^ copper atoms/cm^2^, the charge carrier concentration decreases rapidly. We find that there is a peak in the charge carrier concentration similar to the pure CdTe, but shifted to lower copper concentrations. The mobility values decrease in the range of (0.5–2)×10^15^ copper atoms/cm^2^ added to the sample surface, with an increase again at lower copper values for *x* = 0.08.

Overall, the resistivity values measured with Hall effect measurements behave like the values measured with TLM, with increasing values for increasing Se content. The mobility is similar for all *x* values, with a minimum value in the range of (0.5–2)×10^15^ copper atoms/cm^2^ added to the surface during the doping step. At copper contents higher than this, the mobility of all samples increases by about an order of magnitude. At copper contents lower than the mobility minimum value, a sharp increase in mobility is observed for the x = 0.08 sample. The majority of copper is segregated at the grain boundaries, where it is involved in passivation of defects. DFT calculations show that both Cu’_Cd_ and Cu^•^_i_ can be formed at the grain boundaries [31]. These defects have the opposite effect on mobility as they have on charge carrier concentration: at small Cu concentrations, the Cu’_Cd_ defect is formed, which appears to have a detrimental effect on mobility. At higher Cu concentrations, the Cu’_Cd_ defect might be saturated and the compensating Cu^•^_i_ defect is formed, increasing the mobility again. However, the carrier transport in solar cells is parallel to the grain boundaries and not perpendicular; therefore, the effect of Cu doping on the mobility deduced from Hall effect measurements is not necessarily relevant for the behaviour of solar cells.

For the investigated range of copper addition, the charge carrier concentration is lower in the CdTe samples that contain Se than in pure CdTe. The *x* = 0 sample shows a clear peak in charge carrier concentration with 1.6×10^15^ copper atoms/cm^2^ added to the sample. The measurements indicate a similar peak in the Se-containing CdTe samples, but shifted to lower amounts of added copper atoms. We assume that this peak in carrier concentration is due to the formation of acceptor defects at low Cu content and additional formation of compensating donors at higher Cu content. However, if the peak values at optimum copper amounts are compared, the carrier concentration for *x* = 0.1 is almost an order of magnitude lower than for *x* = 0. This indicates that either the solubility of Cu is reduced in CdTe_1-x_Se_x_ compared to CdTe, or that the formation energy of the compensating donor Cu^•^_i_ is lower in CdTe_1-x_Se_x_, limiting the net p-type conductivity more than in CdTe.

Hall measurements imply that (in the composition range investigated here) the CdTe_1-x_Se_x_ alloy alone cannot be used to increase the majority carrier concentration and thereby increase the *V*_OC_ of solar cells. Further investigations are needed into the role of other alloys and dopants. Alloying CdTe with Se can, however, be used to locally engineer the band gap and the doping concentration in the absorber of a solar cell, thereby yielding device structures that can not be realised with pure CdTe.

### 3.3. Application in a substrate configuration solar cell

We fabricated solar cells in substrate configuration with energy band gap grading in the absorber layer towards the CdS window layer. To achieve this grading a CdSe layer was evaporated on top of the CdTe layer, and during subsequent CdCl_2_ treatment the CdTe_1-x_Se_x_ alloy was formed. The stack was then doped with 1×10^15^ copper atoms/cm^2^. CdS window layers with various thicknesses (see Table 1) were deposited. The standard procedure for state-of-the-art CdTe solar cells in substrate configuration is to deposit 60 nm CdS, followed by a CdCl_2_ treatment and subsequently a second 60-nm-thick CdS layer. For the samples with reduced CdS thickness, the first CdS layer was deposited with CBD followed by a CdCl_2_ treatment. In this case, no second CdS layer was applied. The cells were finished following the procedure described in the experimental section.

**Table 1.** Performance parameters of CdTe solar cells with and without CdTe_1-x_Se_x_ grading of the absorber towards the CdS window layer, and with varying CdS window layer thicknesses.

Cell	CdSe on top of CdTe	CdS thickness	*V*_OC_	*J*_SC_ (EQE)	FF	Efficiency	Band gap (EQE)
	[nm]	[nm]	[mV]	[mA/cm^2^]	[%]	[%]	[eV]
Standard CdTe	0	120	830 ± 1	18.5 ± 0.5	69 ± 4	10.5 ± 0.1	1.49 ± 0.01
CdTe with thin CdS	0	60	691 ± 1	17.3 ± 0.5	52 ± 4	6.2 ± 0.1	1.49 ± 0.01
With CdTe_1-x_Se_x_	50	60	738 ± 1	22.5 ± 0.7	69 ± 4	11.5 ± 0.1	1.44 ± 0.01
With CdTe_1-x_Se_x_	60	30	710 ± 1	25.6 ± 0.8	67 ± 4	12.2 ± 0.1	1.43 ± 0.01

Alloying at the front surface was chosen in order to have a lower band gap in the space charge region, where good collection is expected, to serve as a proof of concept of the influence of a smaller band gap at the front to enhance the spectral response in the NIR. Considering the results from the Hall effect measurements, the doping concentration is expected to be lower in the CdTe_1-x_Se_x_ layer than in CdTe and the space charge region width is therefore expected to be larger. The consequence is a higher absorption in the space charge region and less absorption in the undepleted (quasi-neutral) region, leading to a better collection of photogenerated charge carriers. With the interdiffusion of a thin CdSe layer and the CdTe absorber, a continuous composition gradient is formed at the front of the absorber. This avoids the formation of a step in the gradients of band gap and carrier concentration, which might occur if a CdTe_1-x_Se_x_ layer were co-evaporated on CdTe.

As reported by Kranz et al., standard processing of CdTe solar cells in substrate configuration requires a thick CdS window layer to avoid *V*_OC_ and fill factor losses due to the formation of pinholes in the CdS layer [32]. By changing the front layer of the absorber, these effects can be mitigated and the CdS layer thickness can be reduced.

**Figure 7.**
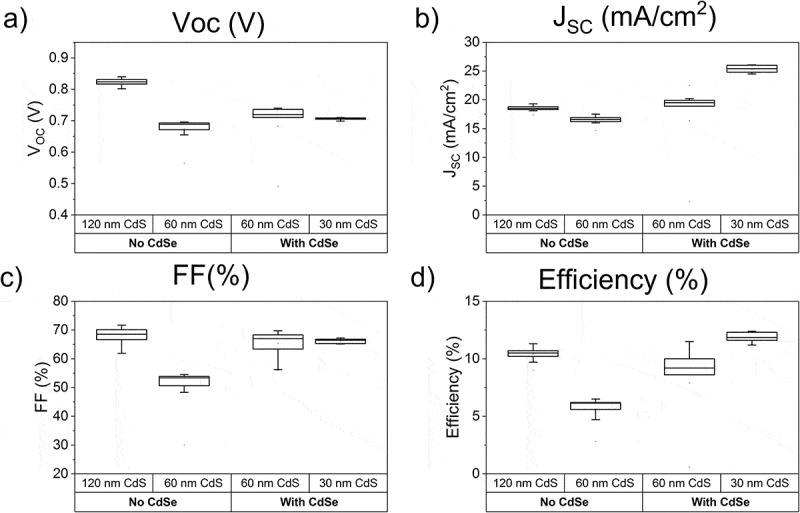
JV performance parameters of CdTe solar cells with and without CdTe_1-x_Se_x_ grading of the absorber towards the CdS window layer, and with varying CdS window layer thicknesses.

**Figure 8.**
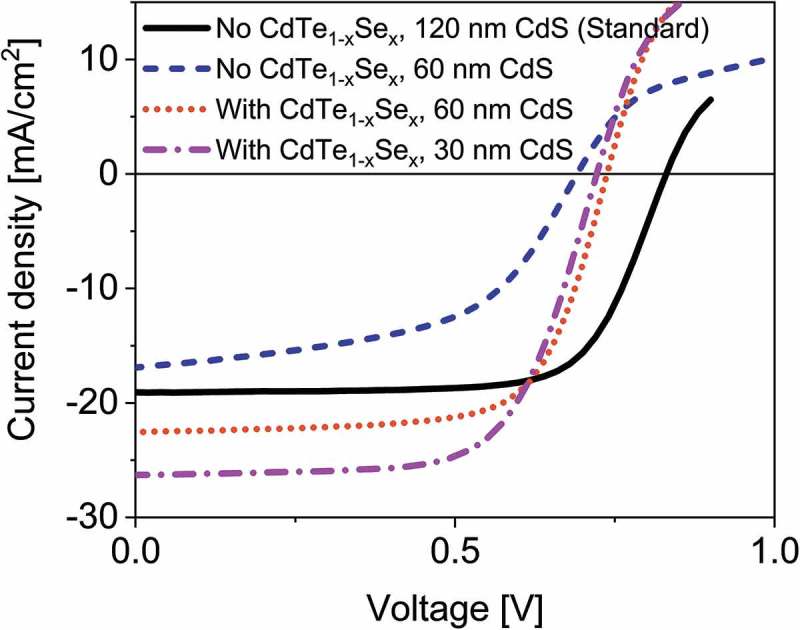
Current-voltage curves of CdTe solar cells with and without CdTe_1-x_Se_x_ grading of the absorber towards the CdS window layer, and with varying CdS window layer thicknesses.

**Figure 9.**
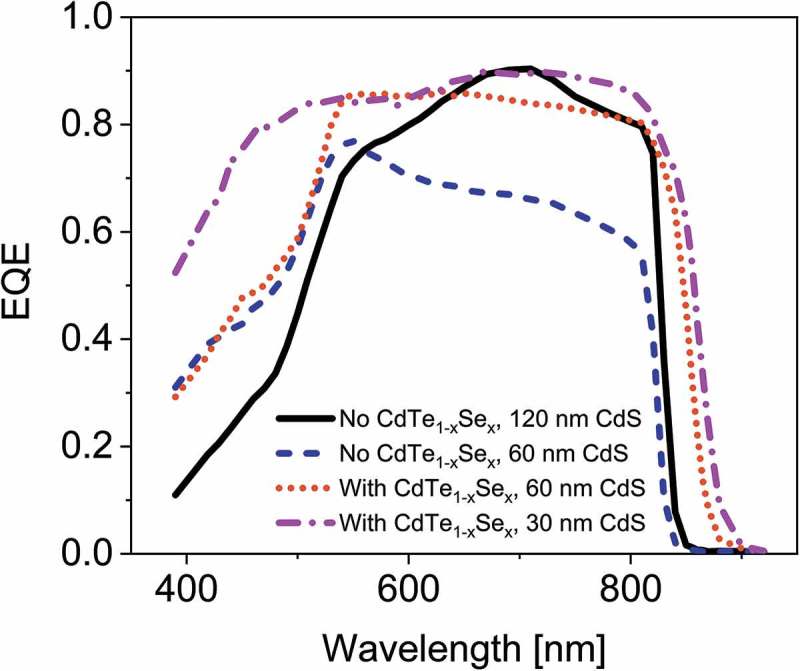
External quantum efficiency of CdTe solar cells with and without CdTe_1-x_Se_x_ grading of the absorber towards the CdS window layer, and with varying CdS window layer thicknesses.

Results from J-V measurements are shown in Figure 7. The values for the best cells are presented in Table 1, with the *J*_SC_ calculated from the external quantum efficiency measurement. The JV and EQE curves of the best cells are shown in Figures 8 and 9. The band gap was calculated from EQE by plotting (hν*EQE)^2^ versus hν and using the intercept of the linear fit with the *x*-axis.

From the band gap, the composition of the CdTe_1-x_Se_x_ alloy can be estimated by using the fit curve from Figure 5. The result is a maximum value of *x* = 0.14 for the sample with 50 nm CdSe and 60 nm CdS, and *x* = 0.17 for the sample with 60 nm CdSe and 30 nm CdS. The charge carrier concentration in the alloy layer is therefore expected to be about an order of magnitude lower than in the CdTe absorber that makes up the bulk of the cell, increasing the width of the space charge region.

The standard CdTe sample shows a low EQE in the short-wavelength region (see Figure 9) due to the parasitic absorption of the double CdS layer. While omitting the second CdS layer reduces this parasitic absorption, it is detrimental to *V*_OC_ and fill factor, as can be seen in the Se-free samples in Figures 7 and 8. Previously reported results show that a single layer of CdCl_2_ treated CdS forms pinholes, giving a direct contact between CdTe and ZnO [32].

In the samples with Se grading at the front surface of the absorber the *V*_OC_ and fill factor values are higher than in the CdTe sample with a single CdS layer, indicating that the problem of pinholes is mitigated. The CdS layer thickness can even be reduced to 30 nm without significant fill factor loss.

The *V*_OC_ is influenced by several factors: We expect the lower band gap and lower carrier concentration in the alloyed absorbers to reduce the *V*_OC_. This can explain the *V*_OC_ difference between the standard CdTe solar cells and the graded absorbers. The *V*_OC_ in the graded absorbers is higher than in the single CdS layer CdTe solar cell, which indicates that the *V*_OC_ loss caused by pinholes in a single CdS layer is diminished.

The short-wavelength region of Figure 9 shows the influence of the CdS thickness and its parasitic absorption on the *J*_SC_. For the cells with a single CdS layer the reduced parasitic absorption compared to the double CdS layer cell results in a *J*_SC_ increase of 1.4 mA/cm^2^. A further reduction of the CdS layer to 30 nm increases the *J*_SC_ in this region by another 1.8 mA/cm^2^.

The long-wavelength region of the EQE shows the *J*_SC_ increase due to the reduced band gap of the CdTe_1-x_Se_x_ alloy. In this region, the *J*_SC_ of the CdTe_1-x_Se_x_ containing solar cells with band gaps of 1.44 eV and 1.43 eV, respectively, are 1.1 and 1.7 mA/cm^2^ higher than the standard CdTe solar cell.

A maximum *J*_SC_ value of 25.6 mA/cm^2^ is achieved, which offsets the losses in *V*_OC_ caused by the grading, resulting in an increased device performance.

## 4. Conclusions

The structural, optical, and electronic properties of the CdTe_1-x_Se_x_ alloy were investigated. The lattice constants of co-evaporated thin films follow Vegard’s law and decrease with increasing *x*. A bowing of the optical band gap was observed with a minimum of 1.40 eV around *x* = 0.3.

We show that alloying CdTe with Se changes the effect of Cu doping on the p-type conductivity. The resistivity of the CdTe_1-x_Se_x_ alloy is higher compared to the CdTe compound, which directly relates to a lower achieved carrier concentration with increasing *x*. In the alloy with *x* = 0.1, the charge carrier concentration is almost an order of magnitude lower than in CdTe. As shown previously, the charge carrier concentration of CdTe is highest with the addition of around 1.6×10^15^ copper atoms/cm^2^. A similar peak in charge carrier concentration is found for the CdTe_1-x_Se_x_ alloy, but shifted to lower copper concentrations.

We fabricated solar cells with compositional, structural, and electronic grading towards the front surface by introducing a CdTe_1-x_Se_x_ layer. The lower band gap in the space charge region results in an increased *J*_SC_ compared to CdTe devices. Additionally, the device performance is improved by a thinner CdS layer. A CdTe_1-x_Se_x_ layer at the CdS/CdTe interface mitigates the formation of pinholes in the CdS layer and the associated losses in FF and *V*_OC_. The parasitic absorption of the CdS is reduced with a thinner layer, also resulting in an increase in *J*_SC_.

These findings show that CdTe_1-x_Se_x_ can be used to fabricate solar cells with increased photocurrent coupled with the possibility of introducing doping gradients into CdTe_1-x_Se_x_ solar cells.

## References

[1] Geisthardt RM, Topic M, Sites JR. Status and potential of CdTe solar-cell efficiency. IEEE J Photovolt. 2015;5(4):1217–1221.

[2] Green MA, Emery K, Hishikawa Y, et al Solar cell efficiency tables (version 46). Prog Photovoltaics. 2015;23(7):805–812.

[3] Zaunbrecher JN, Kuciauskas D, Swartz CH, et al Impact of extended defects on recombination in CdTe heterostructures grown by molecular beam epitaxy. Appl Phys Lett. 2016;109(9):091904.

[4] Sites J, Pan J. Strategies to increase CdTe solar-cell voltage. Thin Solid Films. 2007;515(15):6099–6102.

[5] Perrenoud J, Kranz L, Gretener C, et al A comprehensive picture of Cu doping in CdTe solar cells. J Appl Phys. 2013;114(17):174505.

[6] Wei S-H ZS Chemical trends of defect formation and doping limit in II-VI semiconductors: the case of CdTe. Phys Rev B. 2002;66(15):155211.

[7] Krasikov D, Knizhnik A, Potapkin B, et al First-principles-based analysis of the influence of Cu on CdTe electronic properties. Thin Solid Films. 2013;535:322–325.

[8] Gretener C, Wyss M, Perrenoud J et al. CdTe thin films doped by Cu and Ag-a comparison in substrate configuration solar cells. 2014 IEEE 40th Photovoltaic Specialist Conference (PVSC) IEEE; 2014:3510–3514.

[9] Gessert TA, Wei S-H, Ma J, et al Research strategies toward improving thin-film CdTe photovoltaic devices beyond 20% conversion efficiency. Sol Energ Mat Sol C. 2013;119:149–155.

[10] Swanson DE, Sites JR, Sampath WS Co-sublimation of CdSe_x_Te_1- x_ layers for CdTe solar cells. Sol Energ Mat Sol C. 2017;159:389–394.

[11] Ma J, Wei S-H Bowing of the defect formation energy in semiconductor alloys. Phys Rev B. 2013;87(24):241201.

[12] Poplawsky JD, Guo W, Paudel N, et al Structural and compositional dependence of the CdSe_x_Te_1-x_ alloy layer photoactivity in CdTe-based solar cells. Nat Commun. 2016;7:12537. 27460872 10.1038/ncomms12537 PMC4974465

[13] Santhosh TCM, Bangera KV, Shivakumar GK Synthesis and band gap tuning in CdSe_1-x_Te_x_ thin films for solar cell applications. Sol Energy. 2017;153:343–347.

[14] Sebastian P, Sivaramakrishnan V The growth and characterization of CdSe_x_Te_1-x_ thin films. J Cryst Growth. 1991;112(2–3):421–426.

[15] Paudel NR, Yan Y Enhancing the photo-currents of CdTe thin-film solar cells in both short and long wavelength regions. Appl Phys Lett. 2014;105(18):183510.

[16] Chirila A, Buecheler S, Pianezzi F, et al Highly efficient Cu(In, Ga)Se_2_ solar cells grown on flexible polymer films. Nat Mater. 2011;10(11):857. 21927005 10.1038/nmat3122

[17] Fu F, Pisoni S, Weiss TP, et al Compositionally graded absorber for efficient and stable near-infrared-transparent perovskite solar cells. Adv Sci. 2017;5(3):1700675. 10.1002/advs.201700675 PMC5867048 29593970

[18] Rabadanov MK, Verin IA, Ivanov YM, et al Refinement of the atomic structure of CdTe single crystals. Crystallogr Rep+. 2001;46(4):636–641.

[19] Hodes G, Manassen J, Cahen D Effect of photoelectrode crystal structure on output stability of Cd(Se, Te)/polysulfide photoelectrochemical cells. J Am Chem Soc. 1980;102(18):5962–5964.

[20] Delage C, Carpy A, Goursolle M The TeO_2_-SeO_2_ system: crystal structure of Te_2_Se_2_O_8_ . Cr Acad Sci II. 1982;295(12):981–983.

[21] Žák Z Crystal structure of diselenium pentoxide Se_2_O_5_ . Z Anorg Allg Chem. 1980;460(1):81–85.

[22] Grzechnik A, Farina L, Lauck R, et al Pressure-induced structural deformations in SeO_2_ . J Solid State Chem. 2002;168(1):184–191.

[23] Chate P, Sathe D, Hankare P, et al Synthesis and characterization of cubic cadmium selenide by chemical route. J Alloy Compd. 2013;552:40–43.

[24] Islam R, Banerjee H, Rao D Structural and optical properties of CdSe_x_Te_1-x_ thin films grown by electron beam evaporation. Thin Solid Films. 1995;266(2):215–218.

[25] Lide DR, Haynes WM CRC Handbook of Chemistry and Physics. 90th Edition ed. Boca Raton, FL, USA: CRC Press/Taylor and Francis; 2009.

[26] Yeh C-Y, Wei S-H ZA Relationships between the band gaps of the zinc-blende and wurtzite modifications of semiconductors. Phys Rev B. 1994;50(4):2715. 10.1103/physrevb.50.2715 9976506

[27] Wei S-H, Zhang SB, Zunger A First-principles calculation of band offsets, optical bowings, and defects in CdS, CdSe, CdTe, and their alloys. J Appl Phys. 2000;87(3):1304–1311.

[28] Ninomiya S, Adachi S Optical properties of cubic and hexagonal CdSe. J Appl Phys. 1995;78(7):4681–4689.

[29] Tit N, Obaidat IM, Alawadhi H Origins of bandgap bowing in compound-semiconductor common-cation ternary alloys. J Phys-Condens Mat. 2009;21(7):075802. 10.1088/0953-8984/21/7/075802 21817341

[30] Kranz L, Gretener C, Perrenoud J, et al Doping of polycrystalline CdTe for high-efficiency solar cells on flexible metal foil. Nat Commun. 2013;4:2306. 23942035 10.1038/ncomms3306

[31] Zhang L, Da Silva JL, Li J, et al Effect of copassivation of Cl and Cu on CdTe grain boundaries. Phys Rev Lett. 2008;101(15):155501. 18999610 10.1103/PhysRevLett.101.155501

[32] Kranz L, Schmitt R, Gretener C et al. Progress towards 14% efficient CdTe solar cells in substrate configuration. Proceedings of the 39th IEEE Photovoltaics Specialists Conference; 2013; Tampa, FL, USA., IEEE: 1644–1648.

